# Functional correlates of optic flow motion processing in Parkinson’s disease

**DOI:** 10.3389/fnint.2014.00057

**Published:** 2014-07-08

**Authors:** Deepti Putcha, Robert S. Ross, Maya L. Rosen, Daniel J. Norton, Alice Cronin-Golomb, David C. Somers, Chantal E. Stern

**Affiliations:** ^1^Department of Psychology, Center for Memory and Brain, Boston UniversityBoston, MA, USA; ^2^Athinoula A. Martinos Center for Biomedical Imaging, Massachusetts General HospitalBoston, MA, USA; ^3^Department of Psychology, University of New HampshireDurham, NH, USA

**Keywords:** Parkinson’s disease, fMRI, optic flow, CSv, MT+

## Abstract

The visual input created by the relative motion between an individual and the environment, also called optic flow, influences the sense of self-motion, postural orientation, veering of gait, and visuospatial cognition. An optic flow network comprising visual motion areas V6, V3A, and MT+, as well as visuo-vestibular areas including posterior insula vestibular cortex (PIVC) and cingulate sulcus visual area (CSv), has been described as uniquely selective for parsing egomotion depth cues in humans. Individuals with Parkinson’s disease (PD) have known behavioral deficits in optic flow perception and visuospatial cognition compared to age- and education-matched control adults (MC). The present study used functional magnetic resonance imaging (fMRI) to investigate neural correlates related to impaired optic flow perception in PD. We conducted fMRI on 40 non-demented participants (23 PD and 17 MC) during passive viewing of simulated optic flow motion and random motion. We hypothesized that compared to the MC group, PD participants would show abnormal neural activity in regions comprising this optic flow network. MC participants showed robust activation across all regions in the optic flow network, consistent with studies in young adults, suggesting intact optic flow perception at the neural level in healthy aging. PD participants showed diminished activity compared to MC particularly within visual motion area MT+ and the visuo-vestibular region CSv. Further, activation in visuo-vestibular region CSv was associated with disease severity. These findings suggest that behavioral reports of impaired optic flow perception and visuospatial performance may be a result of impaired neural processing within visual motion and visuo-vestibular regions in PD.

## Introduction

Parkinson’s disease (PD) is a progressive neurodegenerative disorder characterized by tremor, rigidity, postural instability, bradykinesia, and gait disturbance (Young et al., 2010), as well as non-motor dysfunction including perceptual deficits (Davidsdottir et al., 2008; Armstrong, 2011) and visuospatial impairment (Amick et al., 2006; Stepkina et al., 2010; Poletti et al., 2012). Some of the visuospatial impairments seen in PD patients may be related to changes in how egocentric visual motion, or optic flow, information is processed. Identifying changes in the neural basis of optic flow processing in early PD may provide insight into how visuospatial cognition becomes dysfunctional in this disorder.

Optic flow perception has been linked to disturbances in gait (Davidsdottir et al., 2008), heading direction (Chou et al., 2009) and navigational abilities (Young et al., 2010) in PD, and is a critical aspect of visuospatial cognition (Warren et al., 2001). Optic flow paradigms mimic flow field motion as it is experienced in everyday life and include important visual information about our own movement (egomotion) as well as the environment we are moving through (Durant and Zanker, 2012). Prior functional magnetic resonance imaging (fMRI) studies have identified a network of human cortical areas that are responsive to optic flow motion processing, comprising visual motion and visuo-vestibular integration regions. Visual motion areas include the MT complex (MT+; Tootell et al., 1997; Seiffert et al., 2003; Duffy, 2009), area V6, located in the dorsal parieto-occipital sulcus (Pitzalis et al., 2006), and area V3A, which is positioned laterally and inferior to the parieto-occipital sulcus (Tootell et al., 1997; Cardin and Smith, 2010). The optic flow network also includes vestibular regions thought to process visual input, including the parieto-insular vestibular cortex (PIVC) and cingulate sulcus visual area (CSv; Wall and Smith, 2008; Cardin and Smith, 2010). PD pathology impacts optic flow perception at the behavioral level, characterized by veering and navigational difficulty (Davidsdottir et al., 2008; Young et al., 2010), and structurally, as observed in atrophy of regions of the parietal lobe and parieto-occipital sulcus (Tinaz et al., 2011).

We used fMRI to investigate neural correlates of optic flow processing with a focus on previously delineated optic flow processing areas of the brain in PD patients and age-matched healthy control participants (MC). Controlling for local motion energy, we contrasted activation responses during coherent and egocentric flow field visual motion processing from non-coherent, random motion processing, in order to isolate effects from optic flow. Additionally, we examined the ability of participants to determine the direction of coherent motion, and related these findings to PD severity.

## Materials and methods

### Participants

Twenty-four individuals diagnosed with PD and 20 healthy MC adults were initially enrolled. One MC participant was excluded because neuro-ophthalmological examination revealed advanced glaucoma. Two additional MC participants and one PD participant were excluded on the basis of excess motion in the scanner, resulting in a total of 17 MC (7 men, mean age 62.1 years, 1 left-handed) and 23 PD (12 men, mean age 63.5 years, 3 left-handed). All participants provided informed consent in a manner approved by the institutional review boards at Boston University and Massachusetts General Hospital. All study procedures conformed to ethical standards of experiments with human subjects. PD and MC were matched on age, education, male-to-female ratio and visual acuity, and were screened for neurological and psychiatric illness (Table 1).

**Table 1:** **Participant demographics, perceptual screening, and mood assessment**.

	MC (*N* = 17)	PD (*N* = 23)
Age (years)	62.1 ± 9.3	63.5 ± 5.9
Male/Female	7/10	12/11
Education (years)	16.9 ± 2.2	17.7 ± 2.1
UPDRS Total	—	26.8 ± 11.5
UPDRS Motor	—	15.6 ± 7.5
Levodopa Equivalent Dosage (mg/day)	—	410.6 ± 263.6
Hoehn and Yahr	—	2 (median)
Visual Acuity Near (log transform)	0.06 ± 0.12	0.03 ± 0.27
Visual Acuity Far (log transform)	0.01 ± 0.11	−0.08 ± 0.23
Beck Depression Inventory*	2.2 ± 3.1	6.1 ± 4.7
Beck Anxiety Inventory**	1.9 ± 2.1	5.8 ± 3.8

Values presented in the table are means ± standard deviations, unless otherwise noted. UPDRS: Unified Parkinson’s Disease Rating Scale. * *p* < 0.01, ** *p* < 0.001.

Participants diagnosed with idiopathic PD were recruited from the PD Center at Boston Medical Center. All participants taking anti-parkinsonian medications were scanned at peak “ON” levels of medication, and all neurocognitive testing was done when participants were on medication. All patients were on a combination of levodopa-carbidopa, dopamine receptor agonists, or monoamine oxidase B inhibitors. Four patients were also on antidepressant medication, and two other patients were on anti-anxiety medication as needed. Levodopa Equivalent Dosage was calculated as per convention (Tomlinson et al., 2010) to be 410.61 mg/day on average in the PD group. Individuals met the clinical criteria for mild to moderate PD (Hoehn and Yahr stages I–III) as assessed by the Unified Parkinson’s Disease Rating Scale (UPDRS; Movement Disorder Society Task Force on Rating Scales for Parkinson’s Disease, 2003). The median Hoehn and Yahr staging was 2.0, ranging from 1.0 (unilateral) to 3.0 (moderate bilateral). Average total score on the UPDRS was 26.8 and average motor sub-score was 15.5.

All participants were screened for contraindications to MRI. At study entry, the Mini-Mental State Examination (MMSE) was administered to screen for mental status and rule out dementia; no participant met criteria for dementia (Table 1). All participants received detailed health history screening and a neuro-ophthalmological examination to ensure eye integrity and rule out ocular disease or abnormality. Other exclusionary criteria included coexisting, chronic medical illnesses, use of psychoactive medication besides antidepressants and anxiolytics in the MC group (allowed in PD), history of intracranial surgery or head trauma resulting in a loss of consciousness, and history of drug and alcohol abuse.

### Neuroimaging procedure

**Figure 1:**
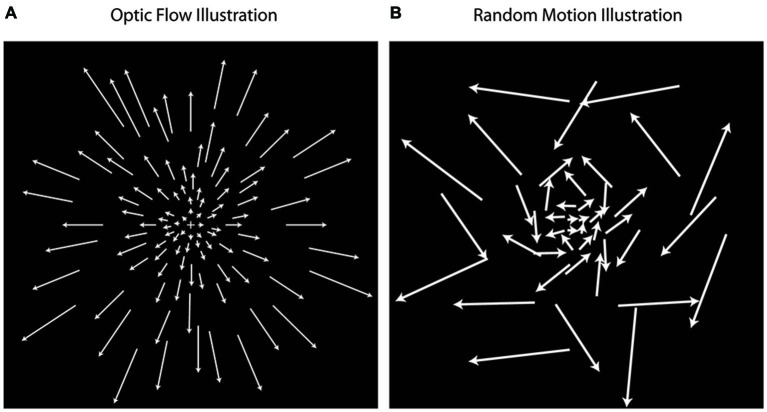
**Optic flow stimuli illustration**. Optic flow motion stimuli **(A)** simulate forward and backward motion using dot fields that are expanding or contracting while rotating about a central focus. Random motion **(B)** simulates non-coherent motion using dots moving at the same speeds used in optic flow, but with random directions of movement. In the illustrations, the length of arrows corresponds with dot speed, indication that dot speed increases with distance away from the center.

Each scanning session included 20 min of structural imaging sequences followed by six runs of the functional optic flow. Each functional run (duration: 4 min 24 s; repetition time = 2 s) consisted of 8 cycles of 16-second alternating blocks of flow motion (“flow”) and random motion (“random”), with the first condition randomly chosen. 2000 moving white dots (each 2 arc-min × 2 arc-min; dot duration 500 ms) were presented within a full screen aperture measuring 10.5° (height) by 16.7° (width). Dot density was 4.14 dots/cm^2^. In both the “flow” and “random” conditions, dot speed scaled with radial distance from the fixation cross (Figure 1). In the “flow” condition, all dots moved with a coherent expansion/contraction direction and/or consistent rotation direction about the central fixation cross (focus of expansion/contraction). In the “random” condition, the dot speed was the same as in the flow condition, but the direction of dot movement was random. In order to drive a maximal number of neurons sensitive to optic flow information, the expansion and contraction of optic flow changed several times per block. There were eight mini-blocks in total throughout each “flow” condition alternating between clockwise and counterclockwise flow during inward and outward contraction/expansion movement of dots, respectively. The direction of dot flow pattern changed between these eight mini-blocks every 500 ms, synchronous with the appearance of a new set of dots (i.e., dot duration = 500 ms). Participants were instructed to relax and maintain fixation in the center of the screen. Visual stimuli were presented with VisionEgg (Straw, 2008). Stimuli were projected from a rear-projection screen and reflected via a mirror that was attached to a 12-channel head coil.

### Image acquisition

Participants were scanned using a Siemens Trio 3T scanner (Siemens Medical Systems, Erlangen, Germany) at the Athinoula A. Martinos Center for Biomedical Imaging. T_1_-weighted Magnetization Prepared-Rapid Acquisition Gradient Echo (MP-RAGE) structural scans were acquired using generalized auto-calibrating partially parallel acquisition (GRAPPA): repetition time = 2530 ms, echo time = 3.44 ms, inversion time = 1100 ms, flip angle = 7°, field of view = 256 mm, slice thickness = 1 mm, 176 sagittal slices (right to left). Blood oxygen level dependent (BOLD) fMRI data during presentation of visual stimuli were acquired using a-weighted gradient-echo echo-planar imaging (EPI) sequence: TR = 2000 ms, TE = 30 ms, FA = 90°, FOV = 256 mm, in-plane resolution 4 × 4 mm^2^. Thirty-two axial (anterior to posterior) slices with a thickness of 4.0 mm were acquired, oriented parallel to the anterior-posterior commissural line. Six functional runs per scan session were acquired, each consisting of 128 whole-brain acquisitions collected immediately after acquisition of four “dummy” TRs for T_1_ stabilization.

### Image processing and analysis

MP-RAGE images were processed using FreeSurfer (version 4.5.0).^1^ Standard preprocessing of structural volumes produced reconstructions that were used to determine if there were any areas of cortical atrophy. The FreeSurfer parcellations were also used to define individual calcarine sulcus labels from which to extract magnitude of activation during the task in each individual’s primary visual cortex. This was done to ensure that activation in the primary visual cortex was comparable between PD and MC groups so that in the event that other cortical activation differences emerged, those results could be interpreted as a result of higher order visual processing. fMRI data were preprocessed using Statistical Parametric Mapping (SPM8; Wellcome Department of Cognitive Neurology, London, UK) for MatLab (The Mathworks, Inc, Natick, Massachusetts, USA). Functional data were re-oriented so that the origin was at the anterior commissure, and standard preprocessing was conducted including motion regression, segmentation, and spatial normalization into standard Montreal Neurological Institute (MNI) space using Diffeomorphic Anatomical Registration Through Exponentiated Lie algebra (DARTEL; Ashburner, 2007). Data were then smoothed using a 6 mm FWHM Gaussian kernel. Quality assurance by motion correction regression was conducted, and time points with >2 mm of motion were modeled out of the analysis, which occurred for less than a third of all available timepoints for only three participants.

Trials were analyzed in a block design format. Conditions were classified as either *flow* or *random* (Figure 1). Following the methods used by Pitzalis et al. (2010), blocks were modeled as 16 s boxcars using the onset of each condition, and *T* contrasts were constructed examining differences in fMRI activation during the *flow* compared to *random* condition within each individual to capture activation response to flow information that was not responsive to random motion. Analysis was based on a mixed-effects general linear model in SPM8.

### Analysis measures

**Figure 2:**
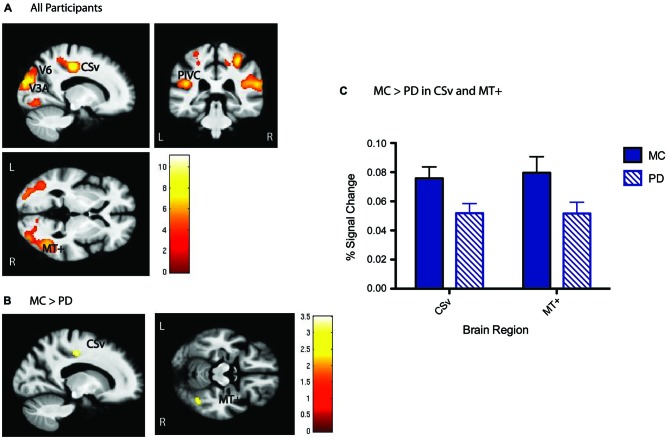
**Optic flow (flow > random) group activation. (A)** Whole group activation in the optic flow network, including V6, V3A, MT+, PIVC, and CSv shown below, *p* < 0.001, cluster corrected with a 46 voxel extent threshold to *p* < 0.01, at MNI *xyz* [−17 −34 0]. Scale bars represent the *t*-statistic. **(B)** Whole brain between group *t*-tests show that CSv and MT+ are significantly more activated in MC than PD, *p* < 0.01 cluster corrected with a 40 voxel extent threshold. Scale bars represent the *t*-statistic. **(C)** Results presented here are another visualization of magnitude of activation presented in **(B)**, depicting higher activation in CSv and MT+ for MC than PD.

Functional whole brain activation maps of *flow vs. random* were generated in all participants together at a voxel activation threshold of *p* < 0.001 cluster corrected to *p* < 0.01 using an extent threshold of 46 contiguous resampled voxels calculated with the AFNI program 3dClustSimm. Next, we performed ROI analysis specific to the optic flow network. Peak activations in our five* a priori* regions of interest (V6, V3A, MT+, CSv, and PIVC) were identified at the whole group level (PD and MC; Figure 2A). Coordinates of peaks are similar to previously published definitions of these regions (Tootell et al., 1997; Cardin and Smith, 2010; Pitzalis et al., 2010): MNI *xyz*: V6: RH[24 −76 32], LH[−20 −78 32]; V3A: RH[22 −84 20], LH[−22 −88 18]; MT+: RH[46 −58 2], LH[−40 −70 6]; PIVC: RH[44 −26 22], LH[−44 −32 20]; CSv: RH[14 −22 48], LH[−12 −20 44]. 5 mm radius spheres around these peak activated voxels were constructed using the MarsBaR toolbox for SPM8. Magnitude of activation defined as percent signal change was then calculated from each of these ROIs. In order to test whether signal measured from the optic flow network ROIs during the *flow* vs. *random* contrast was significantly different for MC and PD, a Group × ROI analysis of variance was conducted on the extracted activation data, followed by post hoc two-sample *t*-tests. All measures of functional activation were averaged across hemispheres for every ROI to create a “bilateral” measure of activation. Next, bivariate correlations yielding Pearson’s product-moment correlation coefficients were used to determine the association between activity in the ROIs and disease severity in the PD participants, as well as between ROI activity and behavioral performance on the coherent motion detection task in MC and PD separately.

### Coherent motion behavioral task

A subset of PD (*N* = 13) and MC (*N* = 12) participants underwent a psychophysical experiment of coherent motion detection. The stimulus was a random dot pattern (RDP) containing signal and noise dots. The signal dots all moved either left or right on any given trial and the noise dots were assigned a random direction at the beginning of presentation and maintained that direction throughout the trial. All dots (200 dots, dot density = 0.97 dots/deg) were white presented on a black background. The dot pattern was presented in a circular window subtending 16.2° of visual angle with the signal and noise dots spatially interleaved. The stimulus was presented for 250 ms on a 21” CRT monitor (Hewlett Packard P 1230) running at 120 Hz at 1024 × 768 resolution. Participants sat 61 cm from the screen. The task was to report the direction of the signal dots (left or right). The stimulus comprised 3, 6, 12, 24, 48, or 96% signal dots with the remainder being noise dots. The main outcome measure of the task was the coherent motion threshold. This was extracted by fitting a Weibull function (Britten et al., 1992) to the accuracy data across the six coherence levels for each participant. The threshold was defined as the minimum coherence level corresponding to 80% accuracy on the Weibull function (Norton et al., 2011).

## Results

### PD is associated with reduced activity in CSV and MT+ during optic flow perception

ROI analysis focused on five cortical regions previously identified as key nodes of the optic flow network in healthy young adults (Cardin and Smith, 2010). These ROIs were defined from the significant activation patterns observed for all participants (Figure 2A). With respect to activation differences during the *flow vs. random* contrast between MC and PD groups, areas CSv and MT+ showed lower activation at the whole brain level (Figures 2B,C). A Group × ROI ANOVA of percent signal change revealed a main effect of group (*F* = 4.23, *dof* = 1, *p* = 0.04), and a main effect of ROI (*F* = 3.12, *dof* = 4, *p* = 0.03). Post-hoc *t-*tests revealed significantly less activation in PD than MC specifically in CSv (*t* = 2.34, *dof* = 38, *p* = 0.02) and MT+ (*t* = 2.13, *dof* = 38, *p* = 0.04). All other regions showed statistically comparable activation between groups: V6 (*t* =1.46, *dof* = 38, *p* = 0.16), V3A (*t* = −0.26, *dof* = 38, *p* = 0.79), PIVC (*t* = 1.29, *dof* = 38, *p* = 0.20). There were no group differences in V1 activity (*p* > 0.9).

### Disease severity is related to activity in CSV

**Figure 3:**
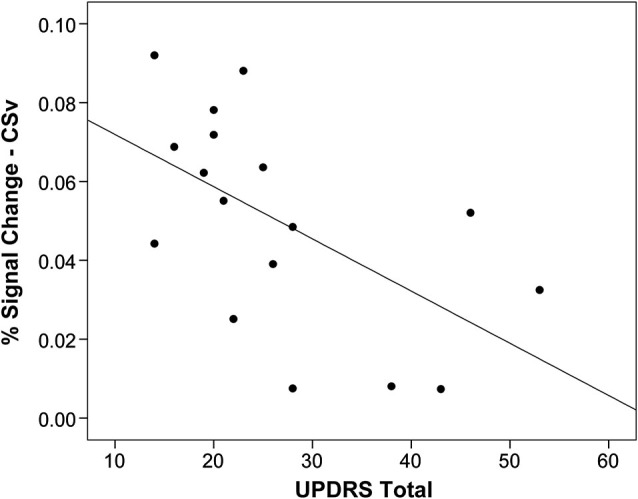
**Activation in CSv related to disease severity**. Activation in response to optic flow in area CSv is correlated with disease severity measured by the Unified Parkinson’s Disease Rating Scale (UPDRS; *r* = 0.56, *p* = 0.02), such that worse disease severity is associated with less activation. Each point on the plot represents one individual with Parkinson’s disease.

Activation in CSv was negatively associated with disease severity within the PD group, as measured by the total UPDRS score (*r* = −0.56, *p* = 0.02), such that more severe disease was correlated with lower activation in this region (Figure 3). There were no significant associations between activation in the other optic flow network regions and disease severity.

### Disease severity is related to coherent motion detection thresholds

In order to assess coherent motion processing behaviorally in PD and MC, performance on the coherent motion task was analyzed for detection threshold at a level of 80% accuracy in each individual. There was no significant difference between MC and PD in detection thresholds (*p* > 0.4), suggesting that the PD group did not differ from MC in low-level motion processing. Within the PD group however, we found a positive association between disease severity and the coherent motion threshold (*r* = 0.69, *p* = 0.01), such that more progressed individuals with PD required higher dot coherency to successfully determine the direction of motion. This suggests that increased severity of PD is associated with impaired low-level motion perception. Coherent motion detection thresholds in the PD group did not correlate with activation in any regions of the optic flow network.

## Discussion

We examined the functional neural correlates of optic flow processing in PD in light of existing behavioral evidence suggesting that disruption in optic flow processing may underlie visuospatial deficits observed behaviorally (Davidsdottir et al., 2008; Young et al., 2010). In healthy young adults, visual cortical areas V6, V3A, and MT+ have been characterized as extracting coherent motion cues selective for self-motion (Cardin and Smith, 2010, 2011; Pitzalis et al., 2010). In addition to these visual motion areas, two regions thought to process and integrate visuo-vestibular input have also been identified as responsive to optic flow stimuli: PIVC and CSv (Wall and Smith, 2008; Cardin and Smith, 2010). In the present study, we established that healthy older adults and individuals with PD also process optic flow in this network of regions similar to what has been observed in young adults. Importantly, we found that individuals with PD showed changes in activation patterns compared to the healthy aging group within the optic flow network overall, and specifically, demonstrated less activity within visual motion area MT+ and the visuo-vestibular region CSv in response to perception of optic flow.

Additionally, within the PD participants, disease severity was correlated with activity in CSv during the optic flow task, further suggesting that this region is targeted by PD pathology. These findings provide new evidence delineating functional neural correlates of disrupted optic flow processing that have been demonstrated behaviorally in PD (Davidsdottir et al., 2008). Further, activity in V1 during this task, together with normal visual acuity, confirmed intact lower-level vision, suggesting that the difference between groups in optic flow processing derives specifically from compromised functioning in regions of the optic flow network. Although the PD participants in this study did not differ behaviorally from MC in the ability to detect direction of coherent motion, success on this task was related to disease severity scores in PD, indicating that within this group of mild-to-moderate PD, those who demonstrated difficulty detecting direction of coherent motion were also those with more severe disease-related impairment. Though it is important to consider the effect of levodopa medication status on these findings, we established that measurements of Levodopa Equivalent Dosage in our PD participants were not significantly related to disease severity or activation in CSv, rendering the drug effect unlikely to fully account for our observations.

Motion information feeds forward from lower-level visual motion areas (V1, MT+, V3A, V6) to more anterior regions of this optic flow network (PIVC and CSv), where vestibular information about body position in space is processed to create an integrated sense of optic flow perception relative to the self (Browning et al., 2009). PIVC and CSv receive inputs from V6 and MT+ (Cardin and Smith, 2010) and activate in response to stimuli consistent with coherent egomotion (Wall and Smith, 2008; Cardin and Smith, 2010). CSv has previously been described as a region that integrates visual input with vestibular signals, and has been implicated in the visual and postural orientation of oneself in the environment (Dean and Platt, 2006; Vogt et al., 2006; Cardin and Smith, 2010; Smith et al., 2012). Individuals with PD have demonstrated difficulty with proprioceptive orientation (Jacobs and Horak, 2006; Vaugoyeau and Azulay, 2010), consistent with our finding of reduced functional activation in CSv in response to optic flow stimuli.

These findings suggest that PD pathology influences optic flow processing even in the mild to moderate stages of the disease. An important caveat is that our PD participants were studied “ON” medication, and it may be of interest to examine participants off medication in future studies. The observed reduction in functional activity during optic flow processing, particularly in CSv, may underlie proprioceptive integration deficits that have been identified as giving rise to later postural instability (Vaugoyeau and Azulay, 2010) and visuospatial dysfunction (Amick et al., 2006; Stepkina et al., 2010; Poletti et al., 2012). Because changes at the neural level often precede striking behavior changes, we postulate that as the disease progresses, the failure to integrate egomotion visual cues with vestibular input in PD leads to a decline in visuoperceptual cognitive function. We suggest that the observed reduction in activation in MT+ and CSv may precede early signs of visuospatial cognitive decline and postural instability as the disease progresses.

## Conflict of interest statement

The authors declare that the research was conducted in the absence of any commercial or financial relationships that could be construed as a potential conflict of interest.

## Footnotes

^1^http://surfer.nmr.mgh.harvard.edu
